# Iron Deposition Leads to Neuronal α-Synuclein Pathology by Inducing Autophagy Dysfunction

**DOI:** 10.3389/fneur.2017.00001

**Published:** 2017-01-16

**Authors:** Wenbin Wan, Lirong Jin, Zigao Wang, Lingyan Wang, Guoqiang Fei, Fanlong Ye, Xiaoli Pan, Changpeng Wang, Chunjiu Zhong

**Affiliations:** ^1^Department of Neurology, Zhongshan Hospital, Fudan University, Shanghai, China; ^2^Department of Neurology, Jingshan Hospital, Fudan University, Shanghai, China; ^3^Experimental Research Center, Zhongshan Hospital, Fudan University, Shanghai, China

**Keywords:** Parkinson’s disease, α-synuclein, iron, autophagy, reactive oxygen species

## Abstract

Growing evidence has indicated that iron deposition in the substantia nigra plays an important role in Parkinson’s disease (PD). However, the underlying mechanism is still elusive. Using primary dopaminergic neurons and SH-SY5Y cells cultured *in vitro*, we observed that iron loading increased α-synuclein and reactive oxygen species (ROS) levels in these cells but did not affect the intracellular α-synuclein mRNA levels. Furthermore, iron loading significantly downregulated Beclin-1 levels and decreased the ratio of microtubule-associated protein 1 light chain 3 isoforms (LC3 II/LC3 I). However, a significant change in the levels of autophagy-related gene 5 (Atg5) was not observed in either neurons or SH-SY5Y cells after iron treatment. After treatment with rapamycin, the iron loading-induced increase in the α-synuclein level was significantly reversed and ROS generation was alleviated in both cultured neurons and SH-SY5Y cells. These results indicate that the inhibition of autophagy is critical for the pathological alterations in α-synuclein induced by iron loading. Moreover, treatment with vitamin E did not affect the increase in the α-synuclein levels but significantly eliminated the iron-induced ROS production. Together, our study shows that autophagy dysfunction contributes to iron-induced α-synuclein pathology.

## Introduction

Parkinson’s disease (PD) is one of the most common movement disorders. PD is clinically characterized by resting tremor, rigidity, and bradykinesia and is pathologically characterized by the aberrant accumulation of α-synuclein and dopaminergic neuron loss in the substantia nigra pars compacta (1–3). The etiology and pathogenesis of PD are still largely unknown, and an effective therapy against disease progression has not been achieved. Thus, more efforts are needed to characterize the pathological etiology of PD.

Since Lhermitte’s first report in 1924 that iron content was increased in the midbrain of individuals with PD (4), the role of iron deposition in PD progression has received substantial attention (5–9). As described in previous studies by our and other groups, an elevated nigral iron level is correlated with the disease severity in patients with PD (7, 10). Accumulating evidence from *in vitro* studies has revealed that the expression of human α-synuclein is modified by iron deposition, which subsequently results in the aggregation and toxicity of α-synuclein (11, 12). Furthermore, an iron chelation treatment was shown to benefit patients with PD; substantia nigra (SN) iron levels were decreased and clinical and radiological improvements were observed (13). Many other studies have also indicated that elevated iron levels in the SN present a tractable target for PD (14, 15). Together, these results indicate that the aberrant accumulation of iron is crucial in PD progression. However, the mechanism underlying the iron-induced development of PD is still elusive.

Oxidative stress has been demonstrated to be an important factor in iron-induced pathologies, serving as a link between iron deposition and PD (16, 17). Elevated iron levels in the SN lead to the generation of reactive oxygen species (ROS), such as superoxide anion radicals and hydroxyl radicals; the increase in ROS level subsequently results in the aberrant upregulation of α-synuclein and damage to dopaminergic neurons in PD (16, 17). In the presence of iron, the highly reactive hydroxyl radical is produced by the Fenton reaction (16). As one of the most harmful ROS, this highly reactive hydroxyl radical damages proteins, nucleic acids, and the lipid membrane, resulting in cell injury and death (16). Nevertheless, controversy still exists. Oxidative stress is not considered a determining factor of the aberrant aggregation of α-synuclein in PD. Radical eliminators thoroughly remove the generated ROS *in vitro* but are unable to entirely reverse the iron-induced upregulation of α-synuclein (18). Moreover, anti-oxidants have little effect on improving the symptoms of PD (19–21). Thus, other potential mechanisms might be involved in the development of PD.

Autophagy is a degradation process that plays a pivotal role in the cellular energy balance and in eliminating misfolded proteins in cells (22). Autophagy is classified as macroautophagy, microautophagy, and chaperone-mediated autophagy (CMA) (23, 24). In the last decade, growing evidence has indicated an intimate relationship between autophagy and PD, showing that autophagy dysregulation may play a critical role in the development of PD. Aggregates of α-synuclein are mainly degraded *via* the autophagy pathway (25–27), including macroautophagy and CMA pathways (24). The autophagy signaling pathway has been reported to be disrupted in PD (28). Furthermore, the inhibition of autophagy results in the pathological accumulation of α-synuclein in neurons (28). However, the role of autophagy in iron-induced PD pathology remains unclear.

In this study, we investigated the effect of iron deposition on neural cells and then evaluated the role of autophagy in the pathological, iron-induced accumulation of α-synuclein.

## Materials and Methods

### Cell Culture and Treatments

SH-SY5Y cells (Type Culture Collection of the Chinese Academy of Sciences, Shanghai, China) were cultured in Dulbecco’s modified Eagle’s medium (DMEM, Gibco, USA) supplemented with 10% fetal bovine serum (Gibco, USA), 100 U/ml penicillin, and 100 µg/ml streptomycin (Gibco, USA) at 37°C in a humidified atmosphere containing 5% CO_2_. The cells were subcultured every 3 days and were grown to 70–80% confluence prior to treatment.

The primary mesencephalic cells were cultured using a previously described method (29) with several modifications. Briefly, on day 17, pregnant Sprague-Dawley (SD) rats were anesthetized, and the fetuses were collected to isolate and digest the cells. The mesencephalic dopaminergic region was retrieved. Cell suspensions were filtered, centrifuged, and plated onto poly-l-lysine-coated dishes at a density of 5 × 10^4^ cells/cm^2^. Approximately 5 h later, the medium was replaced with neurobasal medium supplemented with B27 and GlutaMAX (Invitrogen, USA). The cells were maintained for 12 days before administering the indicated treatment. This procedure used to collect primary cells from SD rats employed in this study was approved by the Medical Experimental Animal Administrative Committee of Zhongshan Hospital, Fudan University.

Cells were treated with ferrous chloride (Sigma, USA) prepared in sterilized water containing 0.01 N HCl as previously described (30). Rapamycin (InvivoGen, USA) was dissolved in DMSO (Sigma, USA) as a 10 mM sterile stock solution, and 0.2 µM was employed as the working concentration of rapamycin, according to the manufacturer’s instructions and previously published work (31, 32). Vitamin E (Sigma, USA) was used as an ROS scavenger as previously described (18, 33). The cells were incubated with the indicated agents for 24 h and then were harvested for detection.

### Knockdown of α-Synuclein with Small Interfering RNAs (siRNAs)

Cholestenone-modified siRNAs targeting rat α-synuclein 5′-CCTCTATGTAGGTTCCAAA-3′ was synthesized by BioTend (China). Twenty-four hours before the iron treatment, the neurons were transfected with the siRNAs using the Lipofectamine^®^ RNAiMAX Transfection Reagent (Invitrogen, USA) according to the manufacturer’s instructions.

### Measurement of Cell Viability

Cell viability was measured using a CKK-8 assay according to the manufacturer’s instructions (Dojindo, Japan). Three hours after the CCK-8 solution was added, the absorbance was determined at 450 nm using a microplate reader (Thermo Fisher, USA).

### Immunofluorescent Staining

Cells were cultured on a round slide. After treatment, the slide was washed with 0.01 M PBS and fixed with 4% PFA as previously described (34). The primary antibody (Mouse anti-α-synuclein: BD, USA; Rabbit anti-NeuN: Abcam, USA) was added and incubated with the cells for 48 h at 4°C. Then, the slide was exposed to the Alexa Fluor^®^ antibody (Invitrogen, USA) and incubated for 1 h at 37°C. The nucleic acids were stained with DAPI (Invitrogen, USA). Following a final wash and mounting with anti-fade medium (Sigma, USA), images were acquired using a fluorescence microscope (Nikon, Japan). The fluorescence intensity was determined using Image-Pro Plus, Version 6.0 (MediaCybernetics, Inc., USA).

### Western Blots

Western blotting was conducted as previously described (35, 36). After an incubation with the indicated antibodies (Beclin-1, Atg5, and LC3 I/II: CST, USA; α-synuclein: BD Biosciences, USA; GAPDH: Santa Cruz Biotechnology, USA), the membranes were analyzed, and images were captured using an Odyssey infrared fluorescence imaging system (LI-COR, USA).

### Real-time qPCR

Quantitative PCR was conducted using our previously described method (36), and the 2^−ΔΔCT^ method was used to analyze the fold change in the levels of the α-synuclein mRNA. The forward and reverse sequences of the PCR primers are listed in Table 1.

**Table 1:** **PCR primers**.

Genes of interest	Gene ID	Sequence (5′ → 3′)	
Human SCNA	6622	Forward	AAGAGGGTGTTCTCTATGTAGGC
		Reverse	GCTCCTCCAACATTTGTCACTT
Human β-actin	60	Forward	CATGTACGTTGCTATCCAGGC
		Reverse	CTCCTTAATGTCACGCACGAT
Rat SCNA	29219	Forward	AAGGGTACCCACAAGAGGGA
		Reverse	AACTGAGCACTTGTACGCCA
Rat β-actin	81822	Forward	CATCCGTAAAGACCTCTATGCC
		Reverse	AGGATAGAGCCACCAATCCAC

### Measurement of Intracellular ROS Levels

The levels of oxidative stress were evaluated using an ROS detection kit (Invitrogen, USA), and the procedure was conducted according to the manufacturer’s instructions. The relative fluorescence intensity of the cells was quantified using a multi-detection microplate reader (Bio-Rad, USA) at an excitation wavelength of 488 nm and an emission wavelength of 525 nm. The intracellular ROS levels were expressed as a percentage of the control cells.

### Transmission Electron Microscopy (TEM)

Transmission electron microscopy was performed using a previously described method (37). Briefly, the cells were pre-fixed with ice-cold 2.5% glutaraldehyde (Sigma, USA) diluted in 0.1 M phosphate-buffered saline and post-fixed with 1% osmium tetroxide buffer. After dehydration in a gradient series of ethyl alcohol, the cells were embedded in epoxy resin. Ultrathin sections (60-nm thick) were stained with uranyl acetate and lead citrate and examined using a transmission electron microscope (Philips CM120, the Netherlands).

### Statistical Analysis

All results are expressed as the means ± SD. Statistical analyses were performed using GraphPad Prism 5.0 software (GraphPad Software, Inc., USA). All experiments were independently repeated three times. The statistical significance of the differences among different groups was analyzed using one-way analysis of variance or Student’s *t*-test, in which *p* < 0.05 was considered significant.

## Results

### Iron Decreased Cell Viability

**Figure 1:**
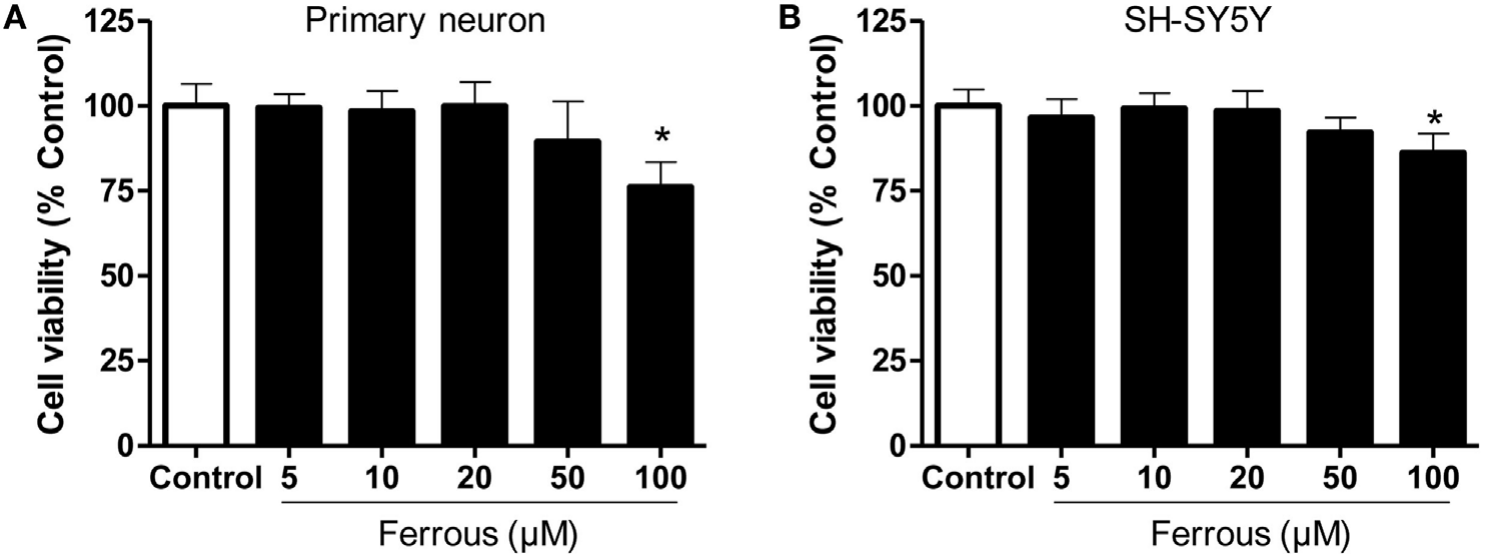
**Effect of iron on cell viability**. The effects of different concentrations of iron on cell viability were determined using the CCK-8 assay. Iron concentrations greater than 50 µM led to significant cell damage. Iron concentrations greater than 100 µM induced marked toxicity to both primary neurons **(A)** and SH-SY5Y cells **(B)**. The results are presented as means ± SD, and one-way analysis of variance was employed to determine the statistical significance of the differences. *p < 0.05 vs. the control.

As shown in Figure 1, primary cultured neurons and SH-SY5Y cells exhibited alterations in cell viability following incubation with different concentrations of ferrous chloride. After a 24-h treatment with 100 µM ferrous chloride, the viability of neurons and SH-SY5Y cells was significantly reduced (*p* < 0.05). The viability of neurons and SH-SY5Y cells exhibited a decreasing trend that was not significantly different (*p* > 0.05) after incubation with 50 µM ferrous chloride. Incubation with 20 µM ferrous chloride did not reduce the viability of neurons and SH-SY5Y cells. Thus, we employed 20 µM ferrous chloride in the subsequent experiments.

### Iron Increased α-Synuclein Levels and ROS Generation but Did Not Affect the Expression of α-Synuclein mRNA

**Figure 2:**
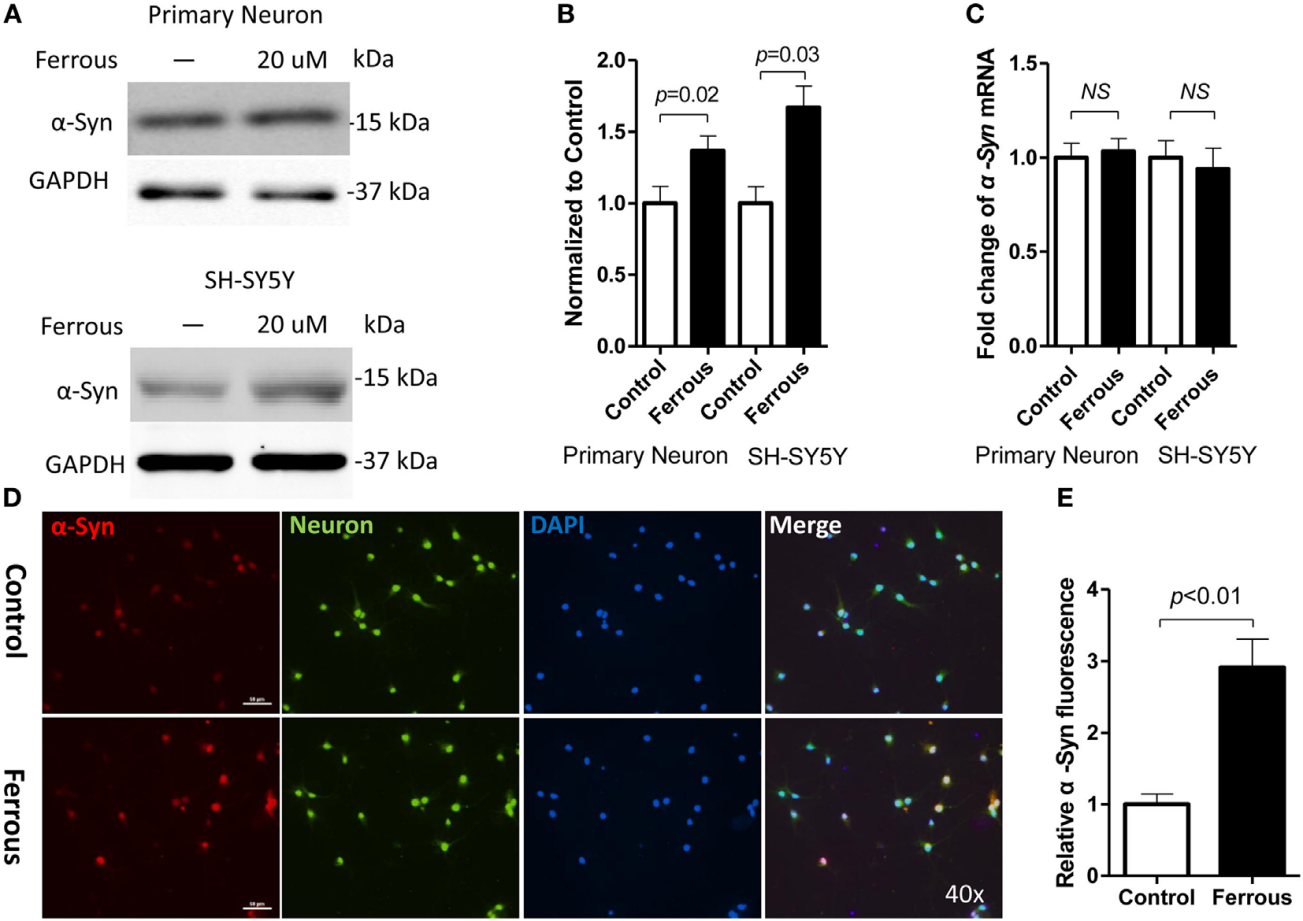
**Iron increased the levels of the α-synuclein protein but did not affect the expression of the α-synuclein mRNA**. After a 24-h iron treatment, cells were harvested to examine the protein and mRNA levels. As shown above, the iron treatment increased the level of the α-synuclein protein [α-Syn **(A,D)**, *p* < 0.05] but did not affect the levels of the α*-Syn* mRNA [**(C)**, *p* > 0.05]. **(B,E)** show the quantitation of the data shown in **(A,D)**, respectively. The results are presented as means ± SD, and Student’s *t*-test was used to determine the statistical significance of the differences.

To determine whether iron deposition affects α-synuclein levels, we evaluated the effect of iron on the changes in the α-synuclein levels in primary neurons and SH-SY5Y cells. Using immunofluorescence staining and Western blotting, we observed that iron exposure upregulated the levels of α-synuclein protein (Figures 2A,B,D,E, *p* < 0.01 for neurons, *p* < 0.05 for SH-SY5Y cells). However, the iron treatment did not affect the levels of α-synuclein mRNA (Figure 2C, *p* > 0.05).

Consistent with the results from previous studies (38), we also observed a 2.6-fold increase in ROS levels in iron-treated cells compared with those of the control (Figure 5A, *p* < 0.05).

### Iron Inhibited Autophagy

**Figure 3:**
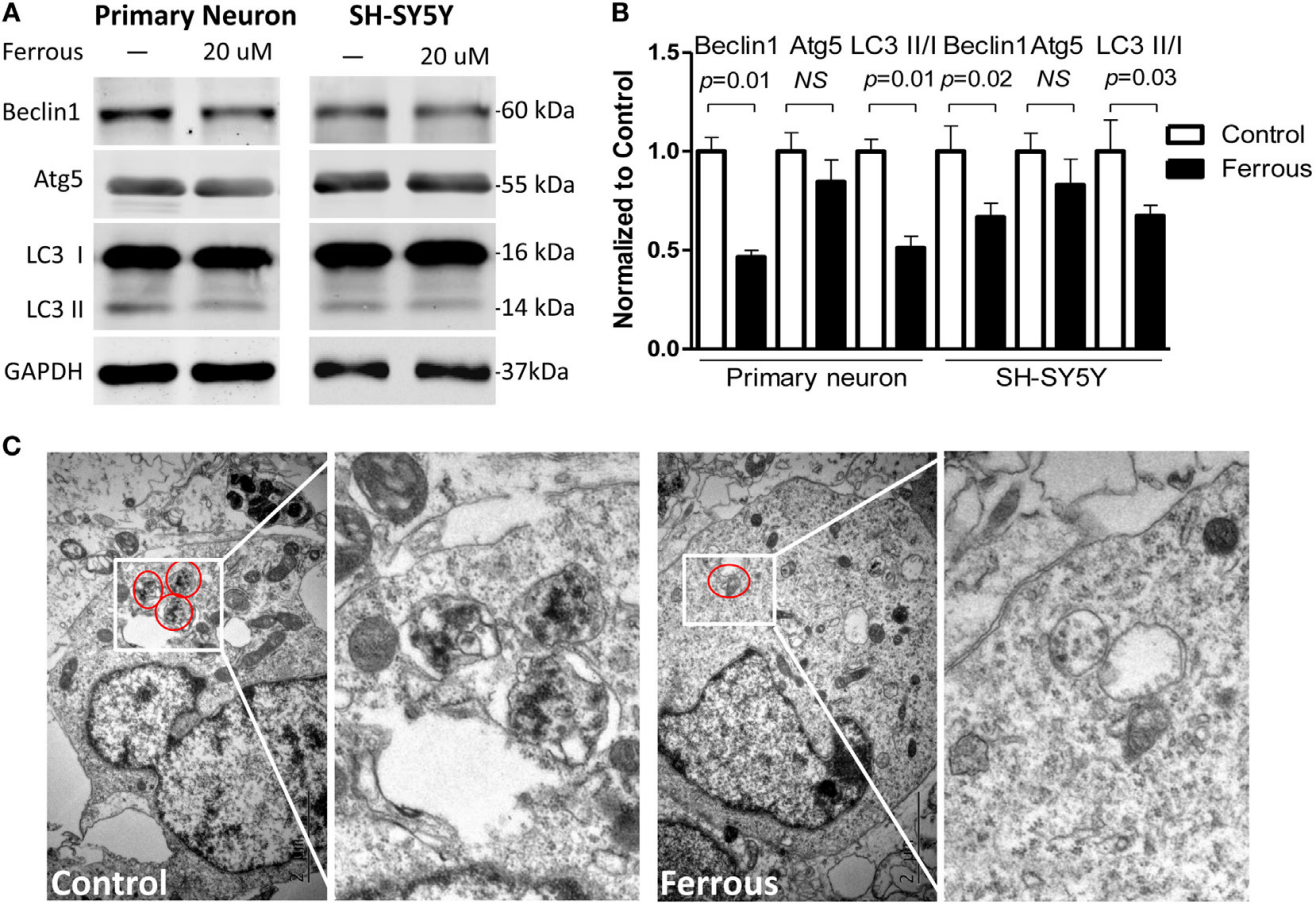
**Iron inhibited autophagy**. The levels of the Beclin-1 (*p* < 0.05) and Atg5 proteins (*p* > 0.05) were decreased in iron-treated cultures **(A)**. The LC3 II level was also decreased and the LC3II/I ratio was reduced in the iron-treated cells [**(A)**, *p* < 0.05]. **(B)** shows the quantitation of the results shown in **(A)**. **(C)** shows the autophagosomes present in primary neurons. The results are presented as means ± SD, and Student’s *t*-test was used to determine the statistical significance of the differences.

Alterations in the levels of the autophagy-related proteins in both of primary neurons and SH-SY5Y cells, including Beclin1, autophagy-related gene 5 (Atg5), and microtubule-associated protein 1 light chain 3 isoforms (LC3 I/LC3 II), were examined by Western blotting. The iron treatment significantly downregulated the levels of Beclin1 (Figures 3A,B, *p* = 0.01 for neurons, *p* = 0.02 for SH-SY5Y cells). Moreover, the levels of LC3 II and the LC3 II/I ratio were also significantly decreased (Figures 3A,B, *p* = 0.01 for neurons, *p* = 0.03 for SH-SY5Y cells). However, Atg5 levels were not significantly reduced in response to the iron treatment (Figures 3A,B, *p* > 0.05).

Using TEM, we consistently confirmed that the number of autophagosomes in the iron-treated neurons was less than the number in control neurons or SH-SY5Y cells (Figure 3C).

### Rapamycin Alleviated Iron-Induced α-Synuclein Accumulation and ROS Generation

**Figure 4:**
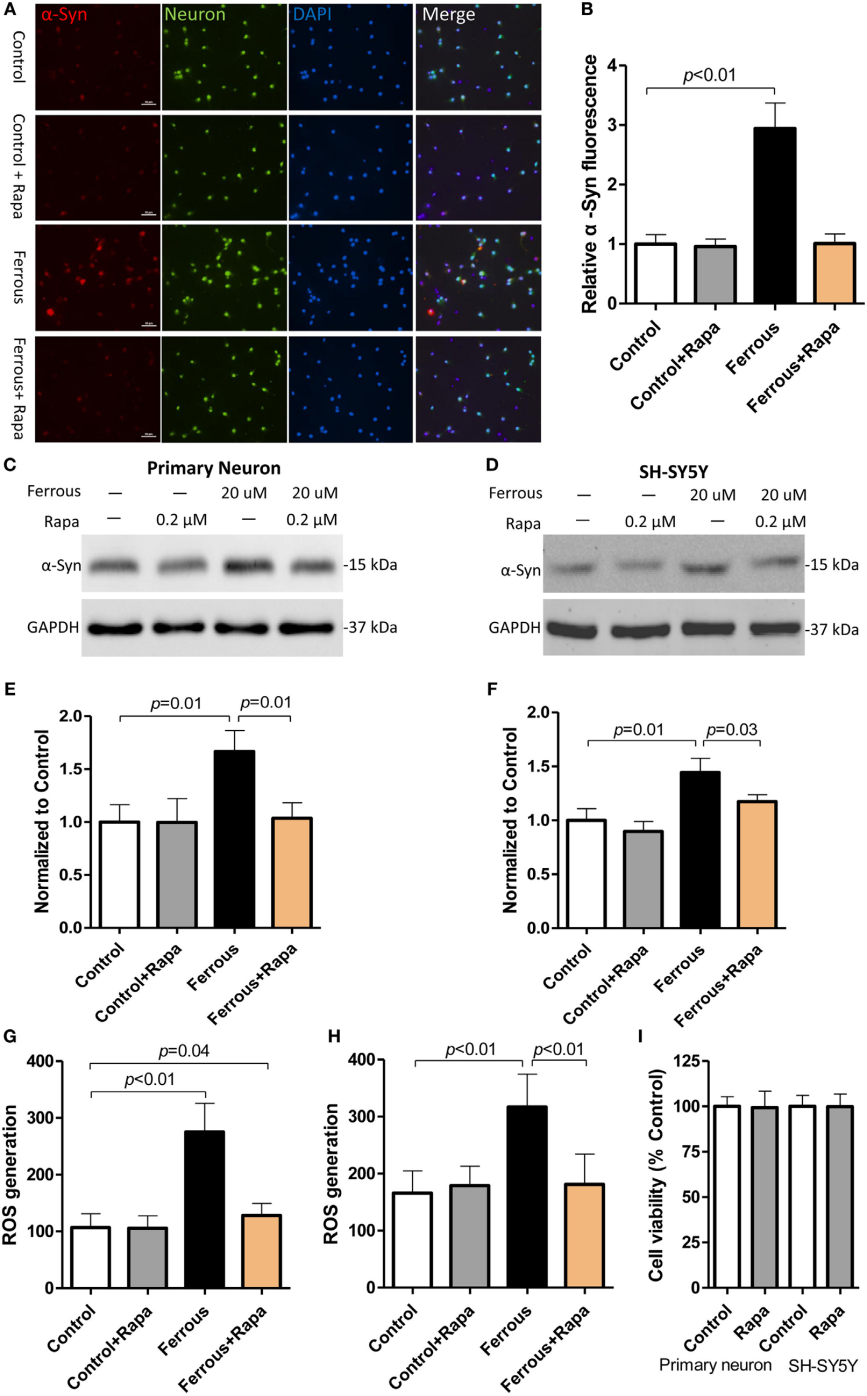
**Rapamycin alleviated the iron-induced pathological changes, including α-synuclein accumulation and reactive oxygen species (ROS) production**. Rapamycin (Rapa) was used to activate autophagy in neurons. Compared with the iron treatment alone, Rapa significantly reversed the upregulation of α-Syn [**(A,C,D)**, *p* < 0.05] and reduced ROS production in the cells [**(G,H)**, *p* < 0.05]. **(A,C,G)** show the data obtained from primary neurons; **(D,H)** show the data obtained from SH-SY-5Y cells. **(B,E,F)** show the quantitation of the data shown in **(A,C,D)**, respectively. **(I)** shows the effect of Rapa on cell viability performing that the concentration of 0.2 µM did not affect cell viability *in vitro* (*p* > 0.05). The results are presented as means ± SD, and one-way analysis of variance was employed to determine the statistical significance of the differences.

To further investigate the role of autophagy inhibition in iron-induced pathological change in our *in vitro* work, we employed an autophagy activator, rapamycin. Following co-incubation with or without iron, rapamycin was observed a slight trend of reduction but did not significantly affect the α-synuclein levels in the control groups (Figures 4A,C,D, *p* > 0.05); however, the upregulation of the α-synuclein levels induced by iron was ameliorated by the rapamycin treatment (Figures 4A,C,D, *p* < 0.05).

We then evaluated the levels of oxidative stress in rapamycin-treated neurons. As shown in Figure 4, rapamycin potently eliminated iron-induced ROS generation (Figures 4G,H, *p* < 0.01) as compared with the iron treatment alone.

### Vitamin E Eliminated Iron-Induced ROS Generation but Did Not Alleviate α-Synuclein Pathology

**Figure 5:**
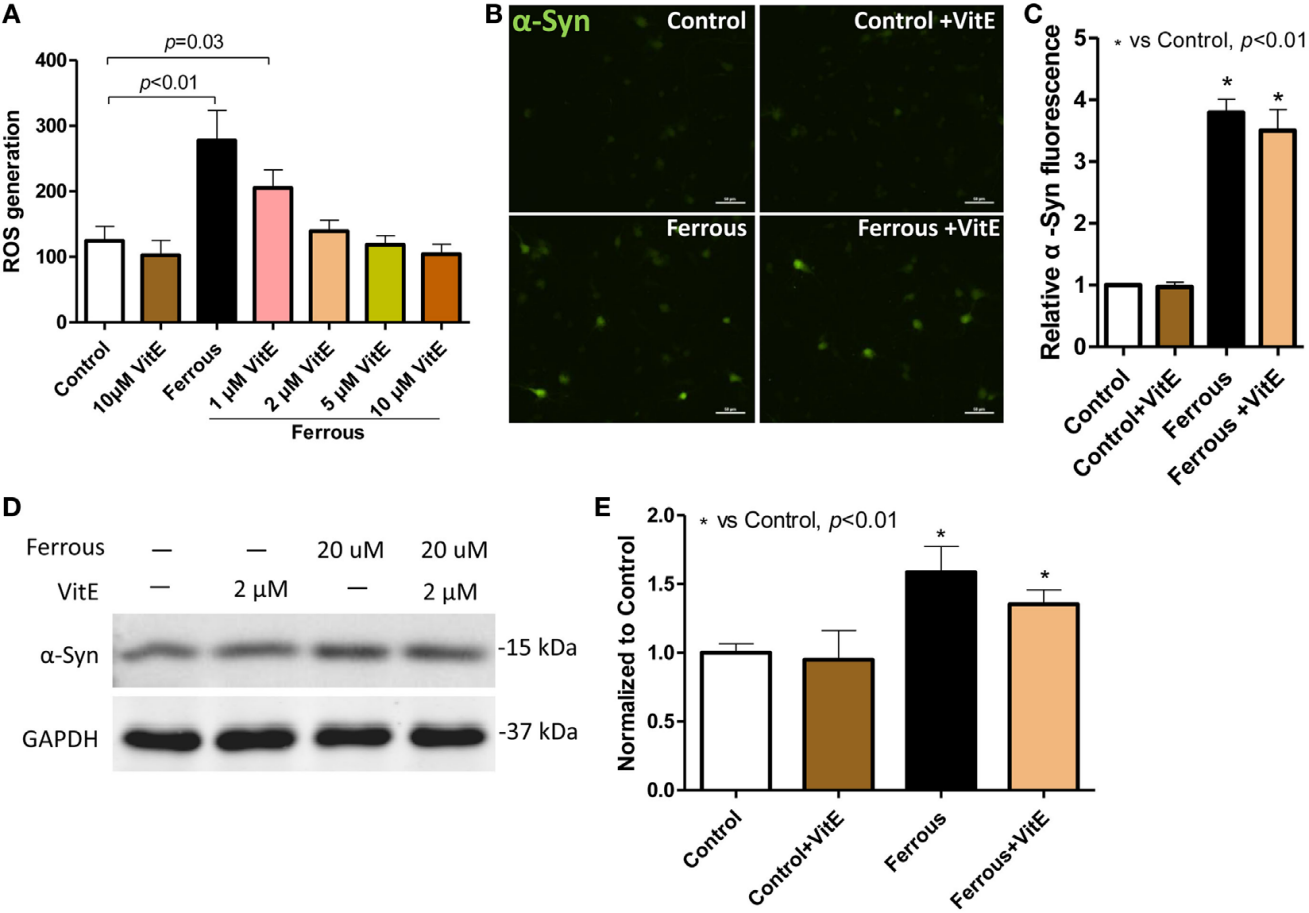
**Vitamin E eliminated reactive oxygen species (ROS) production but did not alleviate iron-induced α-synuclein pathology in neurons**. Vitamin E (Vit E), an ROS eliminator, was added to the cultures to evaluate the role of oxidative stress in iron-induced pathology in neurons. ROS production was completely eliminated by Vit E **(A)**. However, the iron-induced increase in the α-Syn levels was not reduced by the elimination of ROS production **(B,D)**. **(C,E)** show the quantitation of the results presented in **(B,D)**, respectively. The results are presented as means ± SD, and one-way analysis of variance was employed to determine the statistical significance of the differences. **p < 0.05 vs. the control.

As mentioned above, the iron treatment increased the ROS levels *in vitro*, indicating that iron induced oxidative stress. To determine whether the ROS eliminator vitamin E could influence the iron-induced oxidative stress and α-synuclein pathology, we tested the effects of different concentrations of vitamin E on primary neurons. At concentrations ranging from 1 to 10 µM, the vitamin E treatment reduced iron-induced ROS generation in neurons (Figure 5A, *p* < 0.05). In the presence of 1 µM vitamin E, the ROS levels were significantly decreased compared with those of cells treated with iron alone and of the control (Figure 5A, *p* = 0.03). However, following treatment with 2 µM vitamin E, the ROS levels were not significantly different from the control (Figure 5A, *p* > 0.05).

Primary neurons were then treated with 20 µM iron in the presence and absence of 2 µM vitamin E to analyze the effect of oxidative stress on the elevated α-synuclein levels. As shown in Figures 5B–E, incubation with vitamin E alone did not affect the α-synuclein levels in neurons or the iron-induced upregulation of the α-synuclein levels (*p* > 0.05). Although iron-induced oxidative stress was inhibited *in vitro*, the increased α-synuclein levels were not reduced by vitamin E.

### α-Synuclein Silencing Did Not Affect the Iron-Induced Inhibition of Autophagy

**Figure 6:**
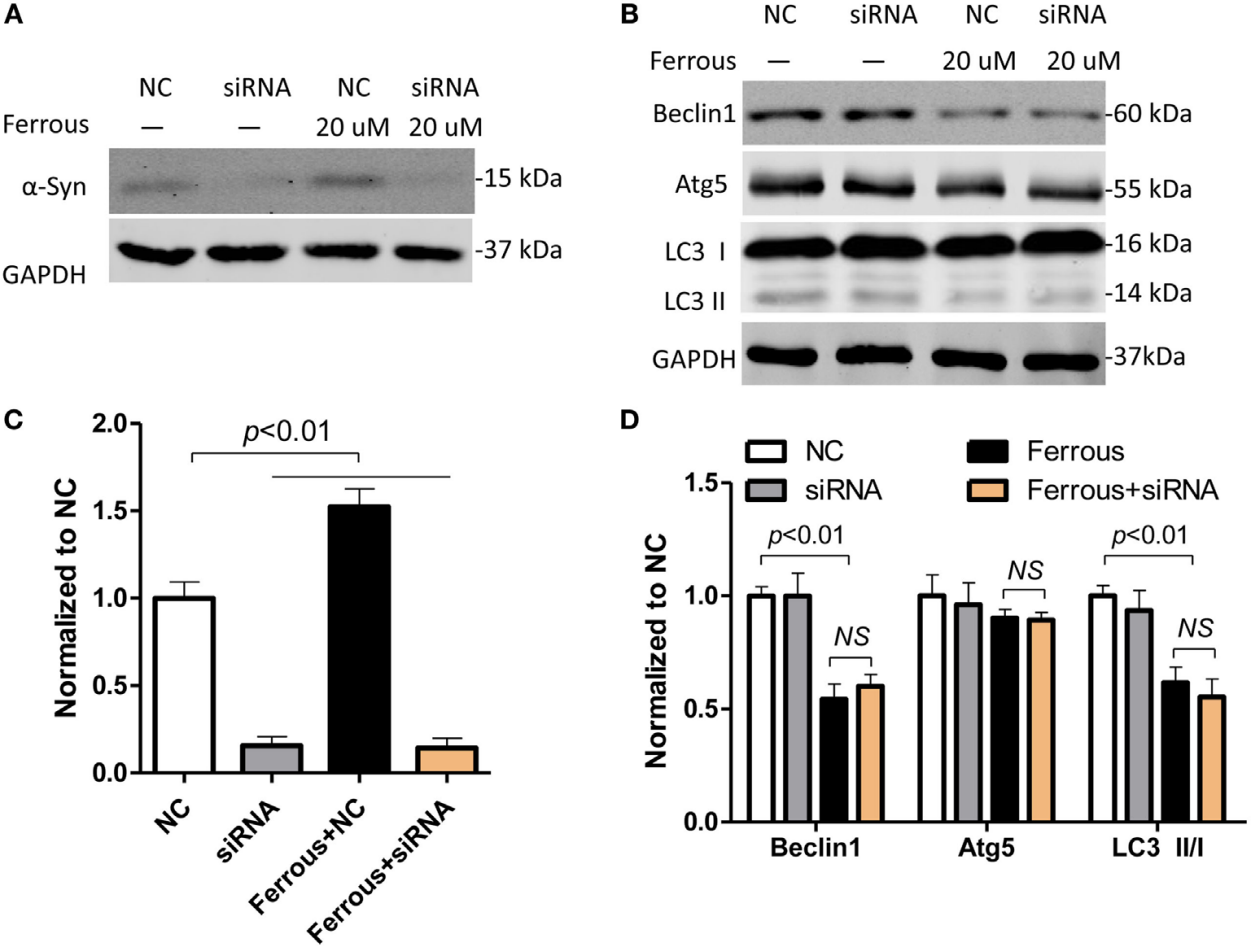
**Iron-induced inhibition of autophagy was not attenuated by α-synuclein silencing in neurons**. The effect of small interfering RNAs (siRNAs) targeting α-synuclein was determined using Western blotting **(A)**. **(B)** shows that silencing of α-synuclein did not affect iron-induced inhibition of autophagy. **(C,D)** show the quantitation of the results presented in **(A,B)**, respectively. The results are presented as means ± SD, and one-way analysis of variance was employed to determine the statistical significance of the differences. **p < 0.05 vs. the negative control (NC).

According to previous reports, the pathological changes in α-synuclein, such as its overexpression and formation of oligomers, inhibit autophagy (39, 40). As shown in this work, iron deposition led to dysfunctional autophagy and α-synuclein pathology. We then transfected neurons with siRNAs targeting α-synuclein to determine whether the inhibition of autophagy was directly mediated by iron or by the increase in the level of the α-synuclein protein. First, the α-synuclein levels were determined in cells transfected with the siRNAs using Western blotting (Figure 6A, *p* < 0.01). Subsequently, we evaluated the changes in the levels of autophagic proteins including Beclin1, Atg5, and LC3 II/LC3 I in iron-treated neurons within siRNAs transfection. The iron-induced downregulation of the Beclin-1, Atg5, and LC3 II levels was not significantly changed by the silencing of α-synuclein compared with the effects seen in the iron-treated neurons (Figures 6B–D, *p* > 0.05).

## Discussion

The mechanisms underlying abnormal α-synuclein aggregation in dopaminergic neurons in patients with PD are elusive and require further investigation. Iron accumulation in the brain has been implicated in PD (5–9), but our current understanding of this phenomenon is very limited. Substantial evidence suggests a robust link between iron deposition in the SN and the pathological lesions observed in patients with PD (11, 12, 14, 15). As shown in this study, iron-treated neuronal cells exhibited α-synuclein aggregates and the autophagy signaling pathway was inhibited. Following an incubation with rapamycin, the iron-induced pathologies of α-synuclein aggregation and ROS generation were alleviated by the activation of autophagy, suggesting a critical role for autophagy in PD.

Oxidative stress results from an imbalance between ROS generation and the intracellular detoxifying systems (16). Oxidative stress has been shown to be an important component of the iron-induced pathological changes, and some researchers posited that oxidative stress is a vital factor that contributes to α-synuclein pathology in PD (16, 17). However, recent evidence has revealed that oxidative stress is not responsible for iron-induced lesions in PD (18), suggesting that the effects of oxidative stress on PD might require re-evaluation. We also achieved a similar result *in vitro* in this study, as the vitamin E treatment eliminated ROS but was unable to reverse the iron-induced upregulation of α-synuclein. Our results are consistent with those of a previously published study by Li et al. who reported that the elimination of iron-induced ROS production only partially alleviated the intracellular α-synuclein aggregation in SK-N-SH cells (18). In previous studies, the administration of anti-oxidant drugs did not confer any protective effects on attenuating the risk of PD (20). Furthermore, a randomized clinical trial showed that a high dosage of anti-oxidants had a finite benefit for patients with PD (19). These results compelled us to examine the mechanisms underlying iron-induced PD-like pathology, in which other mechanisms may be involved.

Autophagy is an intracellular catabolic program that is active under normal and pathological conditions. It is executed by multiple autophagy-related proteins, including Beclin1, LC3I/II, Atg5, and others (41). This process degrades damaged proteins and organelles *via* the autophagy–lysosome pathway and ubiquitin–proteasome system, plays an essential role in the survival of cellular organisms, and provides the necessary materials for cells for cells to compensate for stress conditions such as starvation (41). Excessive activation of autophagy is considered pernicious and has been determined to contribute to neuronal death (42). The disruption of autophagy is also harmful, resulting in the accumulation of misfolded proteins and dysfunctional organelles in cells (43, 44). The coordination of autophagic functions, including activation and inhibition, ensures the balance between cell growth and death. Thus, autophagy is not only critical for cellular survival and normal function but also plays dual roles in cellular life and death (45).

Numerous studies have indicated that autophagy is involved in the pathological changes observed in PD (25–27). However, the explicit role of autophagy in iron-induced pathology is still uncertain. As shown in this study, iron led to α-synuclein pathology in neurons by disrupting Beclin-1-dependent autophagy. Although numerous signaling pathways, including oxidative stress, have been shown to contribute to the progression of PD, the pathogenesis of the disease remains to be clarified. Impaired autophagy has been shown to be involved in the pathological changes in α-synuclein in PD (46–48). As shown in this study, iron loading resulted in α-synuclein accumulation and impaired autophagy in both primary neurons and SH-SY5Y cells. The iron treatment significantly decreased the levels of the Beclin1 and Atg5 proteins. In addition, the LC3 II level and LC II/I ratio were significantly decreased. Rapamycin significantly reversed the iron-induced upregulation of the neuronal α-synuclein levels by activating the autophagy pathway. Furthermore, rapamycin also ameliorated iron-induced ROS generation in neurons, indicating that disruptions in autophagy were involved in iron-induced oxidative stress. Additionally, α-synuclein silencing in primary neurons transfected with siRNAs did not affect the iron-induced inhibition of autophagy, suggesting that the iron treatment primarily disrupted autophagy but not the subsequent pathological changes in the α-synuclein levels. Together, our data indicate that iron-induced autophagy dysfunction may be responsible for α-synuclein accumulation. Moreover, iron deposition alone may directly inhibit the autophagy signaling pathway. However, we still cannot conclude that the pathological changes in α-synuclein observed in this work do not affect the function of the autophagy pathway because the toxic effects of α-synuclein, including the inhibition of autophagy, might be masked by the changes induced by the iron treatment.

In conclusion, autophagy is critical for the iron-induced pathogenesis of PD. Oxidative stress is also associated with α-synuclein accumulation but may not play the key role in iron-induced α-synuclein pathology in PD. Normally, autophagy is an important contributor to intracellular homeostasis; thus, maintaining proper activity of the autophagy pathway is essential for eliminating aberrant protein aggregates. Based on the data from this study, the regulation of autophagy is a potential therapeutic target for PD. However, further investigation is still needed to clarify the role of the autophagy pathway in PD, particularly in *in vivo* models and in patients.

## Author Contributions

LJ and CZ designed the research; WW, ZW, LW, and FY performed the research; XP, CW, and GF analyzed the data; WW and LJ wrote the paper. All the authors approved the final version of the manuscript.

## Conflict of Interest Statement

The authors declare that the research was conducted in the absence of any commercial or financial relationships that could be construed as a potential conflict of interest.
